# Amlou inspired spread: Formulation and characterization of new spread based on *Ziziphus lotus* L. fruit, argan oil, and honey

**DOI:** 10.1016/j.heliyon.2024.e34002

**Published:** 2024-07-02

**Authors:** Hasna Ait Bouzid, Abdelghani Ait Nouisse, Otmane Hallouch, Abderrahim Asbbane, Hicham Harhar, Jamal Koubachi, Filippo Maggi, Giovanni Caprioli, Abdelhakim Bouyahya, Said Gharby

**Affiliations:** a Biotechnology, Analytical Sciences and Quality Control Team, Polydisciplinary Faculty of Taroudant, University Ibn Zohr, Agadir, Morocco; b Laboratory of Materials, Nanotechnology, and Environment, Faculty of Sciences-Rabat, Mohammed V University in Rabat, BP 1014, Rabat, Morocco; c Chemistry Interdisciplinary Project (ChIP) Research Center, School of Pharmacy, University of Camerino, Camerino, Italy; d Laboratory of Human Pathologies Biology, Faculty of Sciences, Mohammed V University in Rabat, Rabat, BP 1014, Morocco

**Keywords:** Amlou, Argan oil, Honey, Antioxidant activity, *Ziziphus lotus* L

## Abstract

This study explores novel applications of combining natural products by integrating *Ziziphus lotus* L. (*Z. lotus*), honey, and argan oil to create a product similar to traditional Moroccan Amlou (a mixture of almonds, honey, and argan oil). Five formulations were developed with varying percentages of these three ingredients, alongside two formulations of traditional Amlou. The nutritional value, mineral composition, fatty acid profile, bioactive compounds, and antioxidant activities of the products were analyzed using standard analytical methods such as gas chromatography and spectrophotometry. Additionally, sensory evaluations were conducted to assess consumer preferences. The results showed that the new formulations are rich in oil (45.15–52.24 g/100 g), carbohydrates (40.26–46.81 g/100 g), and protein (3.15–3.92 g/100 g). Mineral analysis revealed significant amounts of potassium (443–578 mg/100 g), calcium (98–124 mg/100 g), phosphorus (50–65 mg/100 g), and magnesium (38–50 mg/100 g). The *Z. lotus*-based products exhibited higher phenolic content (7–12 mg GAE/g), flavonoids (7.10–10.18 mg QE/g), and stronger antioxidant activities using DPPH radical scavenging activity (3.55–11.14 mg AAE/g) and FRAP (5.39–8.55 mg AAE/g). Moreover, the new product retains the beneficial fatty acid profile of argan oil, with a high content of oleic acid (48 %) and linoleic acid (32 %). Sensory evaluation indicated that the formulation consisting of 45 % *Z. lotus* powder, 50 % argan oil, and 5 % honey was the most appreciated for taste and texture. These findings suggest that incorporating *Z. lotus* into traditional Amlou recipes not only enhances nutritional and antioxidant properties but also meets consumer acceptance in terms of flavor and texture.

## Highlights

• Innovative product development by combining *Z. lotus*, honey and argan oil.
• The obtained formulations displayed high nutritional content of oil.
• The obtained formulations presented important content of total polyphenols.
• The obtained formulations displayed good antioxidant potential.

## 1 Introduction

Researchers have been working to create new functional ingredients that can be added to products as a result of growing customer demands for safe, high-quality foods [1]. Because of this, current research is focused on examining natural sources, like edible wild fruits. These fruits are a source of bioactive components, such as vitamins, carotenoids, alkaloids, carbohydrates, phenols, flavonoids, triterpenoids, saponins, steroids, and tannins [2,3]. They can be incorporated into different rich recipes like spreads. Spreads are nutritious foods that play an important role in the nutrition of humans They are confectionery products made from powdered sugar, vegetable fat, cocoa powder, powdered milk, and other ingredients like nuts [4]. *Ziziphus lotus* L. (*Z. lotus*) or wild jujube is a wild plant species in the Rhamnaceae family, which includes around 170 species of Ziziphus worldwide, and only and only two species are found in Morocco *Ziziphus spina-christi* and *Z. lotus* [5]. It is commonly called Sedra, Zerb, Azzougar, or Tazouggart. The fruit is a drupe called “Nbeg” [6,7]. *Z. lotus* has a wide ecological and geographical distribution in arid and semi-arid plateau regions [8]. This plant has significant bioactivities, including antioxidant, antimicrobial, hepato-nephroprotective, antihyperlipidaemic, anti-inflammatory, analgesic, antidiabetic, and antiproliferative properties [[9], [10], [11]]. *Z. lotus* fruits are known for their high content of total sugars, which account for 88 % of their composition. In addition to being rich in sugars, these fruits also contain large quantities of essential mineral elements such as calcium, potassium, phosphorus, magnesium, and iron, among others. However, they are relatively low in protein, at just 3.56 % [6,12]. These fruits are a rich source of vitamins such as vitamin C, thiamine, biotin, pyridoxine, and vitamin A [13]. Jujubes have been widely exploited in various fields such as nutrition, health, and cosmetics. Due to the popularity of their edible fruits, numerous research studies have been carried out on their use [6]. Indeed, several different products have been created from jujubes, such as jam, syrup, liqueur, bread, additives, and flavorings. In addition, a cake named “Sponge cake” and biscuit have been developed using jujube powder as a partial replacement for flour [14,15].

Among Moroccan local products, Amlou is a traditional Moroccan culinary specialty from the Souss region, and one of the country's most prized local products [16]. It's a highly nutritive spread preparation made from three ingredients namely; Argan oil, roasted sweet almonds, and honey [17]. 100 g of Amlou provides around 690 kcals, 8.7 g of protein, 67 g of fat, 23 g of carbohydrates, 111 mg of calcium, and 108 mg of magnesium [18]. Currently, other altered Amlou products are found in the Moroccan market such as Amlou using argan oil, peanuts, and honey or Amlou using sunflower or soybean vegetable oil, peanuts, and honey [16]. The main objective of this study was the formulation of a new spread using *Z. lotus* fruits, argan oil, and honey. The different formulations were analyzed for their proximate composition, mineral profiling, and antioxidant activity. Alongside sensory analysis was evaluated to determine the appreciation of the products.

## 2 Material and methods

### 2.1 Plant material

*Z. lotus* fruits were harvested at full maturity from the Taroudant region, Morocco in November 2022. After drying, the fruits were ground and sieved. Argan oil samples were prepared in the woman cooperative of Tiout (Taroudant), and Almonds and Honey were purchased from the woman cooperative Tighanimine (Agadir).

### 2.2 Product formulation

The product is prepared using three key ingredients: *Z. lotus* fruit powder, argan oil, and orange honey. To optimize our product and find the ideal formula, five different formulas were developed. At the same time, we also prepared two formulas of Amlou, to compare our creations (Table 1). Contrary to Amlou, no roasting for *Z. lotus* fruits is needed. The choice of the percentages of *Z. lotus* powder was based on preliminary compositions tested for their texture. This explains the use of a higher content of oil than *Z. lotus* powder because the product lost the original Amlou texture.

**Table 1:** Composition of different formula of the product (%).

Product code	*Z. lotus* powder	Almond	Argan oil	Honey
ZL1	48	–	52	00
ZL2	45	–	50	05
ZL3	45	–	48	7
ZL4	42	–	48	10
ZL5	40	–	45	15
AM1	–	80	16	4
AM2	–	76	20	4

### 2.3 Microbial analysis

The microbial analysis was evaluated according to the standard methods described by Zbadi et al. (2015) [19]. Five bacteria were analyzed: Fecal coliforms (FC) (NM 08.0.124 (2012)), total coliforms (TC) (NM 08.0.114 (2008)), Salmonella (NM 08.0.116 (2006)), total aerobic mesophilic flora (TAMF) (NM 08.0.121 (2006)) and Anaerobic sulphite-reducers (ASR) (NM 08.0.125 (2004)).

### 2.4 Proximate composition and energy value

#### 2.4.1 Oil yield

The products were subjected to a cold process extraction using *n*-hexane solvent [20]. The product and *n*-hexane are mixed in a ratio of 1:4 (weight/volume) and stirred in the dark at room temperature for 24 h. After the 24 h extraction period, the mixture was centrifuged at 3500 rpm for 20 min. The solvent (*n*-hexane) was removed by evaporating it under vacuum conditions using a rotary evaporator (R-200, Buchi, Zurich, Switzerland) at 40 °C.

#### 2.4.2 Moisture, ash, and protein content

Moisture content (MC) was measured using a Memmert model oven (Schwabach, Germany). 5 g of sample powder was dried at 103 °C until it reached a constant weight. The moisture content was calculated using the following formula (1):

(1) $$MC\;\left(\%\right)=\frac{P2-P3}{P2-P1}\times 100$$

Where P1 is the initial weight of the crucible, P2 is the weight of the crucible with the sample before drying, and P3 is the weight of the crucible with the sample after drying. The difference in weight was used to determine the water content.

Ash content (AC) was assessed using a muffle furnace (Nabertherm GmbH, Germany). 5 g of developed product was incinerated for 4 h at 525 °C, and the resulting ash was weighed.

Crude protein content (PC) was calculated from the nitrogen (N) content. The N content was determined using a LECO model elemental analyzer (LECO FP628, USA). The measured N was then converted to PC, expressed as a percentage, using a factor of 6.25 [21].

#### 2.4.3 Carbohydrates content (CC)

Carbohydrates content was calculated by subtraction of the sum of MC, PC, AC, and OC values from 100 as follows [22] (2):

(2) CC(g/100g)=100−(%MC+%PC+%AC+%OC)

#### 2.4.4 Energy value (EV)

The energy value (EV) of a food is the amount of energy it provides to our body to maintain our vital functions and carry out our daily activities. It is measured in kilocalories (kcal) or kilojoules (kJ) and depends on the amount of carbohydrates, protein, and fat contained in the food. Here, it is expressed in kcal/100 g of product using the equation below (3) [6].

(3) EV(Kcal/100g)=2.62×PC+8.37×OC+4.2×CC

### 2.5 Mineral determination

Mineral analysis was carried out using a PerkinElmer model Optima 8000 DV inductively coupled plasma optical emission spectrometer (ICP-OES). In summary, 1 g of the product was subjected to treatment in a muffle furnace at 500 °C for 2 h, then, the ash obtained was mixed with 4 mL of 65 % NHO_3_ and 10 mL of HCl and injected into the apparatus. The analysis included ten minerals (Na, Fe, Mn, K, Ca, P, Cu, Mg, B, and Zn) according to the parameters and emission lines as described by Ibourki et al. (2022) [23].

### 2.6 Fatty acid determination

The fatty acids (FAs) composition was determined according to the standard analytical method ISO 12966–2: 2017 [24]. (FAs) were converted to fatty acids methyl esters by refluxing 60 mg of oil with 0.3 mL of 2 M methanolic potassium hydroxide solution for 10 min. Following the mixture's cooling to room temperature, 2 mL of hexane was added, and distilled water was used to wash it. Fatty acid methyl esters (FAMEs)-containing hexane layer has been collected and analyzed by gas chromatography using an Agilent 6890 GC system (USA, Santa Clara) on a CPWax 52 C B column (60 m × 0.25 mm i. d., 0.25 μm film thickness). Helium (flowing at a rate of 1 mL/mn) was used as the carrier gas. The oven, injector and detector were set at temperatures of 185, 200, and 230 °C respectively. The samples were injected in split mode with an injection volume of 1 μL (split ratio). The results were expressed as a relative percentage of the area of each fatty acid methyl ester.

### 2.7 Phenolic compounds content and antioxidant activities

#### 2.7.1 Extract preparation

The extraction of bioactive compounds from the products was performed by macerating the product in methanol (1 g/10 mL) for 24 h with continuous stirring. A Whatman filter was used to filter the mixture, and the extract solution was stored in dark bottles at 4 °C until use.

#### 2.7.2 Total phenolic content (TPC)

TPC was conducted following the method of Folin–Ciocalteu reagent as described by Ait Bouzid et al. (2023) [25]. 0.5 mL of diluted extract was mixed with 2.5 mL of Folin–Ciocalteu reagent (10 %). 4 mL of 7.5 % sodium carbonate (Na_2_CO_3_) solution was added. The mixture was stirred and incubated for 30 min in a water bath at 45 °C. The absorbance of each sample was measured using a UV–Vis spectrophotometer at 765 nm. The results were expressed as mg gallic acid equivalents per gram of product (mg GAE/g P).

#### 2.7.3 Total Flavonoid Content (TFC)

TFC was determined using the aluminum chloride method [26]. First, into a 10 mL volumetric flask, we introduce 1 mL of a diluted extract. Next, we add 4 mL of distilled water and 0.3 mL of 5 % sodium nitrite (NaNO_2_). The mixture was allowed to react for 5 min, then 0.3 mL of a 10 % aluminum chloride (AlCl_3_) solution was introduced and left for 6 min. Next, 1 mL of 2 M NaOH was added and the total volume was adjusted to 10 mL with distilled water. After incubating the solution for 30 min at room temperature, the absorbance was measured at 415 nm using a UV–Vis spectrophotometer. The results were expressed in milligrams of quercetin equivalents per gram of product (mg QE/g P).

#### 2.7.4 DPPH free radical scavenging activity

DPPH free radical (2-2-diphenyl- 1-picrylhydrazyl) was used to determine the antioxidant activity of our products [27]. 0.5 mL of an ethanol solution of DPPH (0.2 mM) was added to 2.5 mL of diluted product extract. After incubation in the dark, at room temperature for 30 min, the absorbance was measured at 517 nm. The percentage of inhibition (RSA: radical scavenging activity) was calculated using equation (4):

(4) %RSA=[(AC−AS)/AC]×100

AC: Absorbance of the control (0.5 mL DPPH and 2.5 mL ethanol).

AS: Absorbance of the sample.

The results were expressed as in milligrams of ascorbic acid equivalents per gram of dry matter (mg AAE/g P).

#### 2.7.5 ABTS radical scavenging activity

Trolox equivalent antioxidant capacity is based on reduction of ABTS^+^ radical. 10 mL of 2 mM ABTS (2,2'-azino-bis (3-ethylbenzothiazoline-6-sulfonic acid)) and 100 μL of 70 Mm potassium persulphate K_2_S_2_O8 in H_2_O were allowed to stand at room temperature and in the dark for 16 h. ABTS solution was diluted in ethanol until an absorbance of 0.700 ± 0.003 at 734 nm. Next, 200 μL of the product's diluted solution and 2 mL of the resultant solution were allowed to react. The reaction mixture was vortexed and after 10 min the absorbance was measured at 734 nm [25]. The results are expressed as mg trolox equivalents per gram product (mg TE/g P).

#### 2.7.6 Ferric reducing antioxidant power (FRAP)

The ability of our products to reduce iron (III) was measured using the FRAP method [28]. 1.25 mL of potassium phosphate buffer (0.2 M, pH 6.6) and 1.25 mL potassium ferricyanide K_3_Fe (CN)_6_ (1 %) was added to 0.5 mL of the diluted extract. The mixture was allowed to react for 20 min at 50 °C, then 1.25 mL of trichloroacetic acid CCl_3_COOH (10 %) was added and the mixture was centrifuged at 3,000 rpm for 10 min. Finally, 1.25 mL of the supernatant was taken to be added to the same volume of distilled water and 0.25 mL of ferric chloride FeCl_3_ (0.1 %). The absorbance of each sample was measured at 700 nm. Ascorbic acid was used as the standard in the preparation of a calibration curve, and the results were reported as milligrams of ascorbic acid equivalents per gram of dry matter (mg AAE/g P).

### 2.8 Sensory analysis

Sensory analysis is defined as “a scientific discipline used to evoke, measure, analyze and interpret responses to the characteristics of foods and materials as perceived by the senses of sight, smell, taste, touch and hearing [29]. As part of this sensory evaluation, a group of 15 panelists (5 women and 15 men) were involved in this study. The products developed were coded in such a way as to preserve the confidentiality of their ingredients and ensure an objective assessment. Sensory analysis was conducted at room temperature under white light [30]. Panelists were asked to evaluate the hedonic attributes (taste, odor, color, and texture) using a 5-point ranking hedonic scale ranging (1: Unpleasant, 2: Slightly unpleasant, 3: Slightly pleasant, 4: Pleasant, 5: Very pleasant).

### 2.9 Statistical analysis

All determinations and measurements were performed in triplicate. Values were represented as means ± SD (standard deviations). Principal component analysis (PCA) was carried out on mean values of parameters of studied products and various. All statistical analyses were done using R software version 4.2.2 and Origin software 2018.

## 3 Results and discussion

### 3.1 Microbial analysis

Microbiological characteristics of different products, specifically in terms of microbial counts, absence of certain pathogens (Salmonella), and other indicators are presented in Table 2. The results of microbiological analyses of the total aerobic mesophilic flora (TAMF) of the products show values between 60 and 4.0 × 10^2^ CFU/g. Levels of total coliforms, fecal coliforms, and anaerobic sulphite-reducing bacteria (ASR) are below 10 CFU/g. About pathogenic micro-organisms, the Salmonella analyses revealed the absence of this bacterium in the products analyzed. These values are within regulatory limits according to FDA (2013) [31]. The low levels of bacteria are a sign of the proper conditions and handling preparation of the products resulting in products with high hygienic quality.

**Table 2:** Microbial analysis of the products.

Product	TC (UFC/g)	FC (UFC/g)	ASR (UFC/g)	TAMF (10^2^ UFC/g)	Salmonella
ZL1	<10	<10	<10	2.9	Absence
ZL2	<10	<10	<10	3.3	Absence
ZL3	<10	<10	<10	3.5	Absence
ZL4	<10	<10	<10	4.0	Absence
ZL5	<10	<10	<10	2.4	Absence
AM1	<10	<10	<10	0.6	Absence
AM2	<10	<10	<10	2.1	Absence

TC: Total coliforms, FC: Fecal coliforms, ASR: Anaerobic sulphite reducers, TAMF: Total aerobic mesophilic flora.

### 3.2 Proximate composition

Water or moisture content represents the total amount of water present in a food product; it is a crucial factor in food products, as it can influence several important aspects of food quality, preservation, and safety [32]. The results in Table 3 showed a variation in water content between the different products. *Z. lotus* powder-based products (ZL1 to ZL5) show water content values ranging from 2.30 g/100 g–3.48 g/100 g. On the other hand, Amlou products (AM1 and AM2) had the lowest water content, with values of 1.78 g/100 g and 1.87 g/100 g respectively. Increasing amounts of honey (with 18.55 g/100 g–21.01 g/100 g of moisture) results in a slight increase in water content in the final product [33]. This observation suggests that the incorporation of honey contributes to increasing the water content of products [33]. The low moisture content of the products gives them the ability to be stored safely for a prolonged period, preserving their quality. This makes them an attractive option for consumers and food producers. High moisture content was observed in some functional cocoa-free spread alternatives (chickpea, black rice, carob, doum, date seeds, and beetroot) varying from 22.19 % for Black rice to 23.42 % for carob) [34].

**Table 3:** Proximate composition of formulated products.

Product	MC (g/100 g)	AC (g/100 g)	PC (g/100 g)	OC (g/100 g)	CC (g/100 g)	EV (kcal/100 g)
ZL1	2.30 ± 0.11^d^	1.33 ±0.03 ^bcd^	3.87 ± 0.24 ^a^	52.24 ± 0.08^b^	40.26 ± 0.65 ^b^	616.48 ± 0.89 ^b^
ZL2	2.72 ± 0.00 ^c^	1.37 ± 0.12 ^bc^	3.92 ± 0.02 ^a^	50.70 ± 1.16 ^bc^	41.29 ± 1.84 ^b^	608.05 ± 6.08 ^bc^
ZL3	2.80 ± 0.05 ^bc^	1.07 ± 0.10 ^d^	3.57 ± 0.13 ^ab^	49.57 ± 0.80 ^cd^	42.99 ± 1.52 ^ab^	604.81 ± 3.53 ^cd^
ZL4	3.14 ± 0.06 ^ab^	1.12 ± 0.03 ^cd^	3.42 ± 0.08 ^ab^	47.97 ± 0.05 ^d^	44.35 ± 0.31 ^ab^	596.74 ± 0.42 ^d^
ZL5	3.48 ± 0.07 ^a^	1.41 ± 0.00 ^b^	3.15 ± 0.21 ^b^	45.15 ± 0.21 ^e^	46.81 ± 0.70 ^a^	582.76 ± 0.35 ^e^
AM1	1.78 ± 0.23 ^e^	2.52 ± 0.03 ^a^	3.11 ± 0.13 ^b^	57.19 ± 0.41 ^a^	35.40 ± 1.13 ^c^	635.50 ± 0.58 ^a^
AM2	1.87 ± 0.06 ^de^	2.40 ± 0.03 ^a^	3.06 ± 0.08 ^b^	59.20 ± 0.14 ^a^	33.47 ± 0.44 ^c^	644.10 ± 0.11 ^a^

On each column, mean values marked with different letters [a-e] indicate significant differences (Tukey test, p < 0.05).

MC; Moisture content, AC; Ash content, PC; Crude protein, OC; oil content, CC; Carbohydrates content, EV; Energy value.

Ash content results showed a significant difference between *Z. lotus* products and Amlou products (AM1 and AM2). Amlou products have ash content values of 2.52 and 2.40, respectively, which were higher than the values recorded for the *Z. lotus* products, which range from 1.07 to 1.41 %. Among the *Z. lotus* products, ZL2 and ZL5 have the highest values in terms of ash content. Our results show lower ash content values than those of *Z. lotus* flour biscuit (4.19 g/100 g), and slightly similar compared to a spread consisting of 7.4 % cacao, 10 % milk, and 13 % hazelnut (2.05 ± 0.07 %) (Heba Sayed 2023) and chocolate spread prepared, without or with probiotics and structured triglycerides (2.01–2.08 %) [35].

Plant proteins are playing an increasingly important role as key components of a healthy diet, underlining their growing value for our well-being and their positive impact on the environment [36]. The results of the analysis of the protein composition of various processed products are presented in Table 3, expressed in g/100 g of product. *Z. lotus* fruit-based products have a protein content ranging from 3.15 to 3.92 g/100 g, which is slightly higher than the values recorded for Amlou products made from almonds, which range from 3.06 to 3.11 g/100 g. The protein levels in our products are lower than *Z. lotus* flour-based biscuit (11.25 g/100 g), the spread of hazelnut [37], chocolate [35], and cashew-nut chocolate [38].

**Fig. 1:**
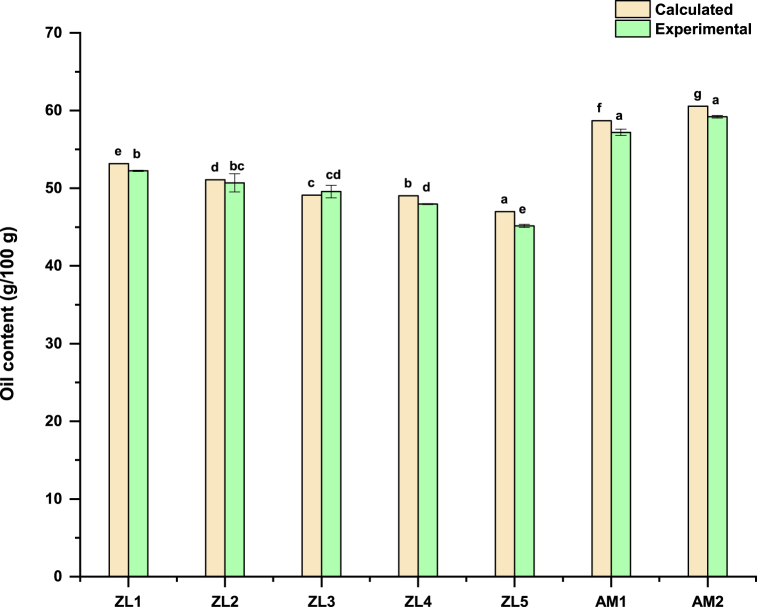
Experimental and theoretical oil content of products. On each oil content (calculated or experimental), different letters [a-g] indicate significant differences (Tukey test, p < 0.05).

The five products based on *Z. lotus* fruit powder have an oil content ranging from 45.15 to 52.24 g/100 g. These values are slightly lower than those obtained for the two Amlou products, which range from 57.19 to 59.2 g/100 g. The high oil content of Amlou products is due to the concentration of oil in the almond kernels, which reached more than 53.37 g/100 g [23] compared to that of *Z. lotus* fruit which was 2.45 g/100 g [6]. *Z. lotus*-based products presented similar oil content as the two formulated spreads prepared using cashew-nut slurry (75 % and 90 %) and cocoa powder (10 % and 25 %) [38]. The results obtained by Nabbouti et al., 2014 [18] for oil content in Amlou (67 g/100 g) correspond similarly to the values measured for our almond-based products (AM1 and AM2). The oil content determined experimentally was compared to that calculated theoretically based on the percentage of argan oil used and the oil content of the *Z. lotus* fruit or almond kernel. The results showed that the oil contents obtained in this study are close to the theoretical values (Fig. 1).

The results of the analysis of total carbohydrates in the processed products are presented in Table 3. Carbohydrate content ranged from 33.47 to 46.81 g/100 g. However, the *Z. lotus*-based products recorded higher values than those for almond-based Amlou (AM1 and AM2), which had values of 35.40 and 33.47 respectively. These results can be interpreted by the high carbohydrate content of *Z. lotus* fruits, which reaches more than 88 g/100 g. Among the *Z. lotus*-based products, ZL5 has the highest carbohydrates value, at 46.81 g/100 g. This value is close to that of chocolate spread with probiotics [35]. On the other hand, ZL1 has the lowest value, at 40.26 g/100 g. This value is close to that of spread prepared by 75 % of cashew-nut slurry and 25 % of cocoa powder [38]. The differences among *Z. lotus*-based products can be interpreted as a function of the amount of honey added to each product. Indeed, product ZL1 contains no honey, while product ZL5 received the highest amount of honey (15 g). The formulations of muskmelon seed and flaxseed multi-nuts spread presented oil contents (43.22 ± 0.44, 44.68 ± 0.39, 43.36 ± 0.50, 45.42 ± 0.53 and 46.64 ± 0.23) in the range of that found in our *Z. lotus*-based products [39].

The food constituents that provide energy are carbohydrates, fats, and proteins. The energy value of foods is usually expressed as a calorific value. Almond-based products (AM1 and AM2) had higher energy values, with averages of 635.50 kcal/100 g and 644.10 kcal/100 g respectively. These values are smaller than those found by Nabbouti et al. (2014) [18] which was around 690 kcal/100 g. In contrast, *Z. lotus* fruit products had slightly lower energy values, ranging from 582.76 kcal/100 g to 616.48 kcal/100 g. These differences can be interpreted by the higher oil content in Almond-based products. In comparison with some chocolate spreads, *Z. lotus* fruit products presented similar energy values (605–612 kcal/100 g) [35].

### 3.3 Fatty acid composition of products oils

The fatty acid composition of the oil extracted from products was analyzed by transforming the fatty acids into methyl esters, and then using gas chromatography to estimate their presence. To facilitate the comparison, the sample of argan oil used in the preparation of these products was analyzed. Table 4 details the fatty acid composition of the different products. *Z. lotus* products had similar values for the major fatty acids, notably oleic acid (48.12–48.69 g/100 g), linoleic acid (32.14–32.51 g/100 g), palmitic acid (13.30–13.78 g/100 g), and stearic acid (4.85–5.31 g/100 g). These values are comparable to those recorded in pure argan oil for the same fatty acids, which are respectively 48.03 g/100 g (oleic acid), 32.45 g/100 g (linoleic acid), 13.36 g/100 g (palmitic acid) and 5.34 g/100 g (stearic acid). Almond-based products were characterized by high oleic acid levels (65.84 and 68.18 ± 0.10 g/100 g), compared to *Z. lotus* products and argan oil. This difference could be attributed to the presence of almonds as an ingredient in almond-based products, which influence fatty acid composition [23]. Almond-based products showed lower levels of linoleic acid, palmitic acid, and stearic acid than pure argan oil. The other fatty acids in Amlou and pure argan oil are present at similar levels and are considered to be present in low concentrations. From these results, we can conclude that *Z. lotus*-based products preserve the argan oil profile while almond-based products preserve the almond oil profile. The argan oil present in our products plays an essential role in extending shelf life, thanks to its antimicrobial properties. Indeed, this oil is known to inhibit the growth of bacteria, fungi, and other micro-organisms that can cause food spoilage [40]. The type of fat used influences the fatty acid composition, for example, the use of ghee or olive oil affects the concentrations of fatty acid in hazelnut spread [41]. It is important to note that solid fats are used to prepare chocolate spreads to give them a creamy taste, spreadability, homogeneity, and stability, but also soybean oil, rapeseed oil, sunflower or olive oils improve the fatty acid content of spreads and had acceptable sensory properties [42].

**Table 4:** Fatty acid composition of products oils.

Fatty acid (g/100 g)	ZL1	ZL2	ZL3	ZL4	ZL5	AM1	AM2	Argan oil	(SNIMA)
C14:0	0.11 ± 0.10^a^	0.09 ± 0.01^a^	0.12 ± 0.10^a^	0.16 ± 0.10^a^	0.11 ± 0.10^a^	0.06 ± 0.01^a^	0.06 ± 0.01^a^	0.09 ± 0.01^a^	≤0.2
C16:0	13.78 ± 0.10 ^a^	13.33 ± 0.10 ^a^	13.40 ± 0.10 ^a^	13.46 ± 0.10 ^a^	13.30 ± 0.10^a^	8.44 ± 0.10^b^	8.87 ± 010^b^	13.36 ± 0.10 ^a^	11.5–15.0
C16:1	ND	0.07 ± 0.01^ab^	0.07 ± 0.01^ab^	0.06 ± 0.01^ab^	0.08 ± 0.01^ab^	0.25 ± 0.10^ab^	0.32 ± 0.10^a^	ND	≤0.2
C18:0	5.05 ± 0.10^a^	4.85 ± 0.10^a^	5.31 ± 0.10^a^	4.99 ± 0.10^a^	5.16 ± 0.10^a^	2.50 ± 0.10^c^	3.12 ± 0.10^b^	5.34 ± 0.10^a^	4.3–7.2
C18:1	48.12 ± 0.10^de^	48.69 ± 0.10^c^	48.25 ± 0.10^cde^	48.55 ± 0.10^cde^	48.64 ± 0.10^cd^	68.18 ± 0.10^a^	65.84 ± 0.10^b^	48.03 ± 0.10^e^	43.0–49.1
C18:2	32.32 ± 0.10^a^	32.51 ± 0.10^a^	32.39 ± 0.10^a^	32.39 ± 0.10^a^	32.14 ± 0.10^a^	20.57 ± 0.10^c^	21.69 ± 0.10^b^	32.45 ± 0.10^a^	29.3–36.0
C18:3	ND	ND	ND	ND	ND	ND	ND	ND	≤0.3
C20:0	0.28 ± 0.10^a^	0.24 ± 0.10^a^	0.21 ± 010^a^	0.17 ± 0.10^a^	0.26 ± 010^a^	ND	0.10 ± 0.01^a^	0.343 ± 0.10^a^	≤0.5
C20:1	0.34 ± 0.10^a^	0.22 ± 0.10^a^	0.23 ± 0.10^a^	0.21 ± 0.10^a^	0.30 ± 0.10^a^	ND	ND	0.39 ± 0.10^a^	≤0.5

On each line, mean values marked with different letters [a-e] indicate significant differences (Tukey test, p < 0.05).

SNIMA: The Moroccan Industrial Standardization Service; ND: not determined.

### 3.4 Mineral content

Elemental analysis is mainly used to determine the mineral content of various samples to show their nutritional composition and check levels of harmful elements [43]. Table 5 shows the average concentrations of various mineral elements analyzed (Ca, Na, K, Mg, Fe, Mn, Cu, Zn, B, and P) in different products. For major elements. Potassium (K) was the most abundant, followed by calcium (Ca), phosphorus (P), and magnesium (Mg). The concentration of Potassium in *Z. lotus*-based products ranged from 4438 for ZL4 to 5786.62 mg/kg for ZL3. Values similar to those observed for Amlou products (AM1 and AM2), which ranged from 3983.54 to 5747.86 mg/kg. For calcium content, *Z. lotus*-based products had concentrations ranging from 984.13 to 1246.38 mg/kg. These values are greater than those found in almond-based Amlou products, which vary from 973.5 to 1072.92 mg/kg. On the other hand, almond-based products have high levels of phosphorus (1204.91–1468.04 mg/kg), magnesium (922.55–972.85 mg/kg) and sodium (40.5–47.37 mg/kg) compared to *Z. lotus* fruit-based products, with values for P (500.99–659.46 mg/kg), Mg (381.91–509.48 mg/kg) and Na (7.77–91.22 mg/kg) respectively. According to the values reported by Nabbouti et al. (2014) [18] for almond-based Amlou, the magnesium content was 108 mg/100 g, while the calcium content was 111 mg/100 g. These values are significantly lower than the results obtained in our study for our products. These notable differences highlight a higher concentration of magnesium and calcium in our products compared to the previous study. Similarly to *Z. lotus*-based products, *Z. lotus* juice contained an important level of calcium compared to date juice [44]. The substitution of cocoa powder with cashew-nut slurry in the spread leads to an increase in magnesium, sodium, and potassium contents [38] but the values are very lower than those for our products. Another study investigates that the fat replaced with eggplant puree in a chocolate spread affected the content of minerals by doubling their concentrations [45]. These spreads contain high levels of minerals compared to our products. For microelements, concentrations are in the following order: iron (Fe), boron (B), manganese (Mn), zinc (Zn), and copper (Cu). Almond-based products stand out for their high concentrations of iron (41.5–48.48 mg/100 g), zinc (10.23–10.59 mg/100 g), and copper (3.9–4.11 mg/100 g) compared with products made from *Z. lotus* fruit. The latter had respective concentrations of Fe (8.13–54.04 mg/100 g), Zn (3.15–4.99 mg/100 g) and Cu (2.48–3. mg/100 g). However, the almond-based products exhibit lower concentrations of boron (12.52–13.8 mg/100 g) and manganese (7.21–7.49 mg/100 g) than the *Z. lotus* fruit products (14.02–24.56 mg/100 g and 9.59–13.9 mg/100 g respectively). Increasing the amount of sugarcane syrup (63 %) with 13 % of sunflower seed of the chocolate spread increased the iron content (11.00–20.34 mg/100 g) [37]. All the formulations with *Z. lotus* or almond except for ZL5 had an important iron content than the spreads formulated by Heba sayed. (2023) [37] and values are above the recommended dietary allowance of iron for adult males, females, and children [37].

**Table 5:** Mineral composition of Z. lotus fruit-based and Amlou products.

Element (mg/kg)/product	ZL1	ZL2	ZL3	ZL4	ZL5	AM1	AM2
Ca	1246.38 ± 0.4 ^a^	1058.59 ± 4.86 ^b^	1231.95 ± 4.15 ^a^	984.13 ± 8.67 ^c d^	995.22 ± 3.06 ^c^	1072.92 ± 1.69 ^b^	973.5 ± 1.68 ^d^
Na	73.86 ± 5.22 ^b^	25.07 ± 0.58 ^d^	7.77 ± 0.75 ^e^	91.22 ± 1.67 ^a^	16.04 ± 2.83 d ^e^	47.37 ± 1.08 ^c^	40.5 ± 1.53 ^c^
K	4471.03 ± 51.25 ^b^	4475.28 ± 97.27 ^b^	5786.62 ± 87.03 ^a^	4438 ± 57.27 ^b^	4445.42 ± 17.7 ^b^	3983.54 ± 24.61 ^c^	5747.86 ± 2.28 ^a^
Mg	435.44 ± 2.6 ^d^	432.42 ± 1.25 ^d^	509.48 ± 4.77 ^c^	381.91 ± 4.57 ^e^	390.15 ± 0.49 ^e^	922.55 ± 6.27 ^b^	972.85 ± 1.8 ^a^
Fe	26.53 ± 1.3 ^d^	54.04 ± 5.64 ^a^	29.11 ± 0.83 ^d^	33.79 ± 1.2 ^c d^	8.13 ± 0.92 ^e^	48.48 ± 2.61 ^a b^	41.5 ± 1.57 ^b c^
Mn	12.37 ± 0.01 ^b^	11.78 ± 0.26 ^b^	13.9 ± 0.16 ^a^	10.01 ± 0.19 ^c^	9.59 ± 0.28 ^c^	7.21 ± 0.19 ^d^	7.49 ± 0.27 ^d^
Cu	3.48 ± 0.03 ^c^	3.03 ± 0.01 ^e^	3.25 ± 0.04 ^d^	2.48 ± 0.02 ^g^	2.7 ± 0.04 ^f^	4.11 ± 0.02 ^a^	3.9 ± 0.03 ^b^
Zn	4.82 ± 0.02 ^b^	4.99 ± 0.04 ^b^	4.73 ± 0.04 ^b^	4.11 ± 0.05 ^c^	3.15 ± 0.01 ^d^	10.59 ± 0.17 ^a^	10.23 ± 0.24 ^a^
B	24.56 ± 0.42 ^a^	15.42 ± 0.15 ^c^	16.58 ± 0.37 ^b^	14.02 ± 0.15 ^d^	14.08 ± 0.28 ^d^	13.8 ± 0.02 ^d^	12.52 ± 0.03 ^e^
P	567.63 ± 0.23 ^e^	595.22 ± 1.91 ^d^	659.46 ± 4.1 ^c^	500.99 ± 4.56 ^g^	529.75 ± 0.53 ^f^	1468.04 ± 0.53 ^a^	1204.91 ± 0.75 ^b^

On each line, mean values marked with different letters [a-g] indicate significant differences (Tukey test, p < 0.05).

Fe; iron, B; boron, Mn; manganese, Zn; zinc, Cu; copper. K; Potassium, Ca; calcium, P; phosphorus, Mg; magnesium, Na; Sodium.

### 3.5 Total phenolic and flavonoids content, and antioxidant activity of the products

The results of bioactive compounds quantification and antioxidant activities of the products are shown in Table 6. Products made from *Z. lotus* fruit show higher total phenolic content ranging from 6.93 for ZL2 to 12.82 mg GAE/g P for ZL1, which is significantly higher than the concentrations observed in Amlou based on almond products, ranging from 2.01 to 2.36 mg GAE/g P. The phenolic content of our products is higher than that of a cake made from *Z. lotus* fruit powder (38.12 mg GAE/100 g). *Z. lotus*-based products are rich in polyphenols as compared to some spreads in the literature. For example, the total phenolic content of the samples formulated using sugarcane, hazelnut or sunflower seed, cacao, and milk ranged from 30.01 to 117.31 mg/100 g [37]. In contrast, the chocolate spread fortified with jackfruit flour presented high total phenolic content (88–127 mg GAE/g) [30]. Products made from *Z. lotus* fruit are characterized by higher flavonoids concentrations, ranging from 7.10 to 10.18 mg QE/g P, compared with Amlou products, which have values ranging from 3.52 to 6.37 mg QE/g P. The flavonoids content of our product is higher than that reported by Najja et al. (2020) [14] for ‘Sponge cake’ made from *Z. lotus* fruit powder (23.13 mg GAE/100 g). This is in line with the results of the comparison of phenolic compounds in *Z. lotus* fruit juice and Dates juice in which juice from *Z. lotus* presented a high content of phenolic compounds [44]. The total phenolic and flavonoids contents in *Z. lotus* products are due to their content in the fruits of *Z. lotus* [6]. The results of phenolic compounds showed that our products contained higher TFC than TPC. This may be explained that the Folin-Ciocalteu reagent used to measure TPC may not react in the same way with all types of phenolic compounds. Some non-flavonoids phenolics may not react or not be detected. In contrast, the aluminum chloride colorimetric method for TFC determination reacts specifically with flavonoids, potentially providing a more accurate representation of these compounds in our samples.

**Table 6:** Total phenolic and flavonoids content, and antioxidant activity of the products.

Produit	TPC (mg GAE/g P)	TFC (mg QE/g P)	DPPH (mg AAE/g P)	FRAP (mg AAE/g P)	ABTS (mg TE/g P)
**ZL1**	12.82 ± 0.24 ^a^	10.18 ± 0.11 ^a^	11.14 ± 2.17 ^a^	7.27 ± 0.09 ^ab^	0.46 ± 0.16 ^c^
**ZL2**	6.93 ± 0.24 ^b^	7.10 ± 1.20 ^ab^	8.73 ± 0.12 ^ab^	5.39 ± 0.18 ^ab^	0.97 ± 0.02 ^b^
**ZL3**	7.32 ± 0.12 ^b^	8.95 ± 1.09 ^a^	13.45 ± 4.54 ^a^	7.68 ± 0.40 ^ab^	0.99 ± 0.11 ^b^
**ZL4**	7.90 ± 0.35 ^b^	7.45 ± 0.49 ^ab^	10.78 ± 1.23 ^a^	8.55 ± 0.49 ^a^	0.89 ± 0.04 ^b^
**ZL5**	7.12 ± 0.31 ^b^	8.25 ± 2.07 ^a^	3.55 ± 1.64 ^b^	7.43 ± 1.81 ^ab^	1.04 ± 0.13 ^b^
**AM1**	2.01 ± 0.31 ^c^	3.52 ± 0.27 ^b^	3.90 ± 1.13 ^b^	4.64 ± 0.62 ^b^	1.63 ± 0.00 ^a^
**AM2**	2.36 ± 0.22 ^c^	6.37 ± 0.05 ^ab^	3.90 ± 0.91 ^b^	3.80 ± 1.64 ^b^	0.76 ± 0.00 ^bc^

On each column. Mean values marked with different letters indicate significant differences (Tukey test. p < 0.05).

TPC: Total Phenolic Content, TFC: Total Flavonoid Content, DPPH: (2-2-diphenyl-1-picrylhydrazyl, FRAP: Ferric reducing antioxidant power, ABTS: 2,2′-azino-bis (3-ethylbenzothiazoline-6-sulfonic acid)).

DPPH assay is widely used in many studies to assess antioxidant activity because it is suitable for different sample types and is sensitive enough to detect active substances even at low concentrations [46]. The results obtained are shown in Table 6. *Z. lotus*-based products showed considerable antioxidant activity with 3.55–13.45 mg AAE/g P compared to those of almond-based products that presented the same values for AM1 and AM2 (3.9 mg AAE/g P). Generally, spreads like our products are known for their antioxidant power, as they include potent ingredients such as nuts [47]. The same trend of results was found for eggplant puree which increased the antioxidant activity of the spread, as it is considered one of the ten best vegetables by its ability to absorb oxygen radicals [45]. ABTS test works by generating a blue-green compound called ABTS^●+^ that can be reduced by antioxidants. It is more effective at detecting highly pigmented and hydrophilic antioxidants than the DPPH assay [48]. The results of antioxidant activity measurements by the ABTS method indicate that *Z. lotus* fruit products (ZL1 to ZL5) have relatively low ABTS values, ranging from 0.46 to 1.04 mg TE/g P. ZL5 has the highest value. In contrast, Amlou's products (AM1 and AM2) have higher ABTS values, with AM1 having a value of 1.63 mg TE/g P, while AM2 has a value of 0.76 mg TE/g P. FRAP test has been used to measure the iron-reducing capacity of various fruits and vegetables, as well as certain biological samples. This test is interesting and potentially beneficial as it can provide a preliminary indication of the presence of antioxidants [49]. The FRAP results show significant differences between *Z. lotus* fruit products (ZL1 to ZL5) and Amlou products (AM1 and AM2). *Z. lotus* fruit products showed higher FRAP values, ranging from 5.39 to 8.55 mg AA/g P, with ZL4 showing the highest value among the analyzed samples. In contrast, Amlou products made from almonds show lower FRAP values, ranging from 3.80 to 4.64 mg AA/g P, with AM2 showing the lowest value. The FRAP assay mainly measures the ability of the sample to reduce ferric ions, which is a specific reaction; but does not necessarily reflect the overall antioxidant capacity of the sample [50].

### 3.6 Sensory analysis

**Fig. 2:**
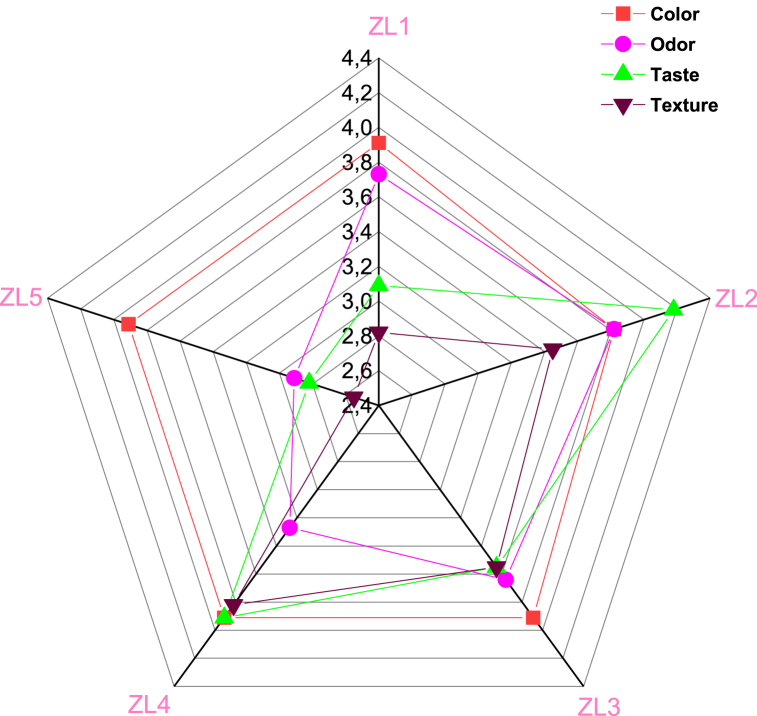
Sensory attributes of developed products.

The results of the sensory analysis can provide important information for improving the formulation and optimizing *Z. lotus-*based products, taking into account consumer preferences. The results are presented in Fig. 2, providing an overview of the sensory evaluations of *Z. lotus*-based products. Concerning color: The products ZL1, ZL2, ZL3, ZL4, and ZL5 obtained similar average color scores, all around 3.9. This indicates a generally positive assessment of the color of the *Z. lotus* products evaluated. ZL2 (45 % *Z. lotus*, 50 % argan oil, and 5 % honey) ranked with the highest score of taste (4.18) and odor (3.82) while ZL5 (40 % *Z. lotus*, 45 % argan oil, and 15 % honey) ranked with the lowest scores (2.82 and 2.91 respectively). The texture of products describes their smoothness, ZL4 (42 % *Z. lotus*, 48 % argan oil, and 10 % honey) had the highest preference with a mean score of 3.82 followed by ZL3 (45 % *Z. lotus*, 48 % argan oil, and 7 % honey) with a score of 3.55, and ZL2 with a score of 3.45. ZL5 presented the lowest scores of odor, taste, and texture.

### 3.7 Principal component analysis (PCA)

**Fig. 3:**
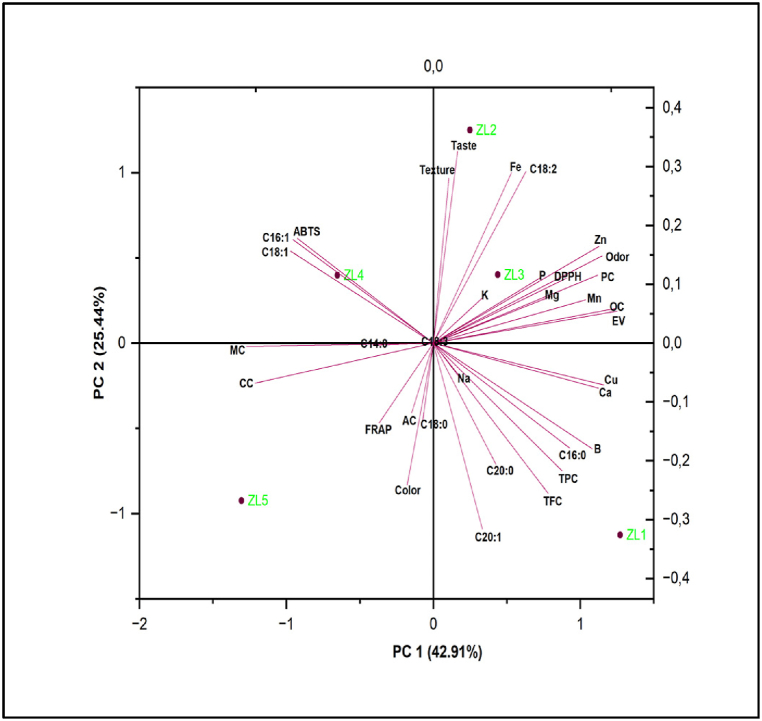
PCA analysis of the studied products.

In this study, PCA was used as a multivariate statistical approach for the possible separation of our developed products (*Z. lotus*-based products) based on the dependent variables studied (Proximate composition, energy value, minerals, fatty acids, TPC, TFC, DPPH, FRAP, ABTS, and sensory attributes). Fig. 3 shows the average values of the various parameters analyzed, as a function of the products developed, and distributed over the surface area produced by PC1 and PC2. The majority of the parameters are distributed in the positive direction of PC1 (42.91 %). ZL2 was associated with the highest values attributes of taste, and texture, iron (Fe), and C18:2, while ZL1 interacted with high values of TFC, TPC, C16:0, C20:0, C20:1, and boron (B). Likewise, ZL3 was marked with a high content of Potassium (K). The product ZL4 was linked to high antioxidant activity ABTS, and fatty acids (C16:1 and C18:2). Finally, ZL5 presented moderate appreciation of color, antioxidant activity FRAP, ash content (AC), and carbohydrates content (CC).

## 4 Conclusion

The development and formulation of a new product based on the fruits of *Z. lotus*, such as Amlou, reveal significant nutritional potential. Our innovative approach combines *Z. lotus* with argan oil, resulting in products rich in essential nutrients, including minerals and antioxidants. In addition, they maintain the argan oil's beneficial profile*.* The sensory analysis showed strong preference for products with higher content of argan oil for its texture and taste. This dual advantage-nutritional benefits coupled with gustatory pleasure-makes our product as an attractive choice for those seeking both gustatory pleasure and nutritional benefits. Our study introduces a novel functional food that could have strong commercial potential.

## Ethics declaration

All participants provided written informed consent to participate in the study and for their data to be published.

## Data availability

Data will be made available on request.

## CRediT authorship contribution statement

**Hasna Ait Bouzid:** Writing – original draft, Methodology, Investigation, Conceptualization, Data curation, Formal analysis, Writing – review & editing. **Abdelghani Ait Nouisse:** Investigation, Formal analysis, Data curation, Methodology. **Otmane Hallouch:** Investigation, Formal analysis, Data curation. **Abderrahim Asbbane:** Investigation, Formal analysis, Data curation. **Hicham Harhar:** Investigation, Formal analysis, Visualization. **Jamal Koubachi:** Investigation, Formal analysis, Visualization. **Filippo Maggi:** Writing – review & editing, Formal analysis, Investigation, Visualization. **Giovanni Caprioli:** Writing – review & editing, Formal analysis, Investigation, Visualization. **Abdelhakim Bouyahya:** Formal analysis, Investigation, Visualization. **Said Gharby:** Project administration, Methodology, Investigation, Data curation, Conceptualization, Resources, Supervision, Validation, Writing – review & editing.

## Declaration of competing interest

The authors declare that they have no known competing financial interests or personal relationships that could have appeared to influence the work reported in this paper.
