# Supplementary material for: Amlou inspired spread: Formulation and characterization of new spread based on Ziziphus lotus L. fruit, argan oil, and honey
Source: Heliyon. 2024 Jul 2;10(14):e34002. doi: 10.1016/j.heliyon.2024.e34002 (PMC11292231; doi:10.1016/j.heliyon.2024.e34002)
Supplement: Multimedia component 1 [file mmc1.docx]

**Tasting Test Questionnary**

In the context of characterizing and formulating a product based on wild jujube (*Ziziphus lotus* L.), you are asked to examine and taste the different formulas, then answer the following questions by checking the box corresponding to your response.

1. **Are you?**

- Female
- Male

1. **How old are you?**

- <20
- 21-30
- 31-40
- >40

1. **Do you consume spreads?**

- Yes
- No

1. **How many times per week do you consume it?**

- Everyday
- 2 to 3 times
- 1 to 2 times

1. **Please examine and taste each sample and indicate how much you liked it.**

| **ZL1** | Unpleasant | Slightly unpleasant | Neither unpleasant nor pleasant | Slightly pleasant | Pleasant | Very pleasant |
| --- | --- | --- | --- | --- | --- | --- |
| **Color** |  |  |  |  |  |  |
| **Odor** |  |  |  |  |  |  |
| **Taste** |  |  |  |  |  |  |
| **Texture** |  |  |  |  |  |  |

| **ZL2** | Unpleasant | Slightly unpleasant | Neither unpleasant nor pleasant | Slightly pleasant | Pleasant | Very pleasant |
| --- | --- | --- | --- | --- | --- | --- |
| **Color** |  |  |  |  |  |  |
| **Odor** |  |  |  |  |  |  |
| **Taste** |  |  |  |  |  |  |
| **Texture** |  |  |  |  |  |  |

| **ZL3** | Unpleasant | Slightly unpleasant | Neither unpleasant nor pleasant | Slightly pleasant | Pleasant | Very pleasant |
| --- | --- | --- | --- | --- | --- | --- |
| **Color** |  |  |  |  |  |  |
| **Odor** |  |  |  |  |  |  |
| **Taste** |  |  |  |  |  |  |
| **Texture** |  |  |  |  |  |  |

| **ZL4** | Unpleasant | Slightly unpleasant | Neither unpleasant nor pleasant | Slightly pleasant | Pleasant | Very pleasant |
| --- | --- | --- | --- | --- | --- | --- |
| **Color** |  |  |  |  |  |  |
| **Odor** |  |  |  |  |  |  |
| **Taste** |  |  |  |  |  |  |
| **Texture** |  |  |  |  |  |  |

| **ZL5** | Unpleasant | Slightly unpleasant | Neither unpleasant nor pleasant | Slightly pleasant | Pleasant | Very pleasant |
| --- | --- | --- | --- | --- | --- | --- |
| **Color** |  |  |  |  |  |  |
| **Odor** |  |  |  |  |  |  |
| **Taste** |  |  |  |  |  |  |
| **Texture** |  |  |  |  |  |  |
